# What drives centralisation in cancer care?

**DOI:** 10.1371/journal.pone.0195673

**Published:** 2018-04-12

**Authors:** Melvin J. Kilsdonk, Sabine Siesling, Boukje A. C. van Dijk, Michel W. Wouters, Wim H. van Harten

**Affiliations:** 1 Netherlands Comprehensive Cancer Organisation, dept. of research, Utrecht, the Netherlands; 2 University of Twente, School for Management and Governance, dept. of Health Technology and Services Research, Enschede, The Netherlands; 3 University of Groningen, University Medical Centre Groningen, dept. of epidemiology, Groningen, the Netherlands; 4 The Netherlands Cancer Institute, Amsterdam, The Netherlands; Centro per lo Studio e la Prevenzione Oncologica, ITALY

## Abstract

**Background:**

To improve quality of care, centralisation of cancer services in high-volume centres has been stimulated. Studies linking specialisation and high (surgical) volumes to better outcomes already appeared in the 1990’s. However, actual centralisation was a difficult process in many countries. In this study, factors influencing the centralisation of cancer services in the Netherlands were determined.

**Material and methods:**

Centralisation patterns were studied for three types of cancer that are known to benefit from high surgical caseloads: oesophagus-, pancreas- and bladder cancer. The Netherlands Cancer Registry provided data on tumour and treatment characteristics from 2000–2013 for respectively 8037, 4747 and 6362 patients receiving surgery. By plotting timelines of centralisation of cancer surgery, relations with the appearance of (inter)national scientific evidence, actions of medical specialist societies, specific regulation and other important factors on the degree of centralisation were ascertained.

**Results:**

For oesophagus and pancreas cancer, a gradual increase in centralisation of surgery is seen from 2005 and 2006 onwards following (inter)national scientific evidence. Centralisation steps for bladder cancer surgery can be seen in 2010 and 2013 anticipating on the publication of norms by the professional society. The most influential stimulus seems to have been regulations on minimum volumes.

**Conclusion:**

Scientific evidence on the relationship between volume and outcome lead to the start of centralisation of surgical cancer care in the Netherlands. Once a body of evidence has been established on organisational change that influences professional practice, in addition some form of regulation is needed to ensure widespread implementation.

## Introduction

Centralisation of low volume cancers and high-risk surgical procedures is a frequently studied organisational quality issue, especially in surgical oncology. The first volume-outcome relationship in surgery was described in 1979 by Luft et al.[1] In the following decades numerous studies have addressed the question whether higher surgical volumes result in an increased quality of care.[2] Many of these studies concerned cancer surgery and a large body of evidence developed in favour of centralisation of surgical procedures such as pancreatectomies and oesophagectomies.[3] In general, a higher volume of surgery is associated with lower post-operative mortality and morbidity.[2, 4, 5] Nevertheless, in the Netherlands, referral patterns for pancreatic and oesophageal cancer remained largely unchanged up to the early 2000’s, despite a lively debate on the introduction of minimum surgical volumes.[6]

There may be several reasons why centralisation was not directly embraced as a method to improve cancer care. The quality of the scientific evidence was questioned as many early studies were observational and not hypothesis driven and few studies actually investigated quality improvement after centralisation.[5] Possible differences in case-mix restricted the generalisability of the available scientific evidence to the Dutch healthcare situation (as most studies were performed in the Unites States). As with any new treatment or technology there is a diffusion period before it becomes widely implemented. For example, a Dutch study on the dissemination of the sentinel node biopsy in breast cancer revealed a gradual increase over the course of 5 years (1998–2003).[7] There is still debate on volume thresholds, ceiling effects and the exact mechanisms through which quality is improved, though at present only a few question the need to centralise low volume and high-risk or complex procedures. Centralisation of services is a delicate issue as professional pride and material interests could play a role in the debate and consequent decisions.

The first Dutch scientific evidence for a positive volume-outcome relationship in pancreas and oesophagus surgery was published by Gouma et al. in 1997 & 2000 and by van Lanschot et al. in 2001.[8–10] Wouters et al. showed reduced postoperative morbidity and mortality after centralising oesophageal resections between 2000–2004.[11] In 2003, the Dutch Healthcare inspectorate started a new supervision policy based on publicly reported quality indicators including total number of surgeries for low volume tumours.[12] The first form of regulation started in 2006 when the Healthcare Inspectorate (IGZ) banned oesophageal resections from hospitals with an annual surgical volume lower than 10. This number was also advised for pancreatic resections but not officially regulated. In 2010 the “quality of cancer care” report was published by the Dutch Cancer Society.[13] In this report, centralisation of low volume tumours and high-risk procedures was regarded to be one of the main strategies to reduce variation in outcome. The Healthcare Inspectorate insisted that in 2011 all medical specialists societies published minimum volume standards (insisting on minimum volumes of 20 operations per year) for highly complicated procedures and regulation would follow from 2013 onwards. In 2011 the Association of Surgeons in the Netherlands (ASN) increased the minimum annual number of low volume, high-risk operations to 20. In 2012, the Dutch Federation of Oncological Societies (in Dutch: SONCOS, consisting of the Dutch Associations for Surgical Oncology (NVCO), Medical Oncology (NVMO) and Radiotherapy and Oncology (NVRO)) set minimum volume standards for the treatment of several types of cancer.[14] In recent years, insurance companies started to use these thresholds for contracting policies adding an extra stimulus to the centralisation debate.

It is unknown whether and which professional, organisational and regulatory stimuli are most effective in stimulating centralisation. Studying this might also provide a more general insight in what drives quality related organisational change in cancer care. We performed a nationwide analysis on the centralisation of oesophagus, pancreas and bladder cancer surgery. Oesophagus and pancreas cancer are the most studied types of cancer in relation to the volume of surgery. Bladder cancer is likely to benefit from centralisation but minimum thresholds were not established in the Netherlands until 2010.[15–18] We hypothesise that even though centralisation of surgery will occur voluntarily and gradually based on scientific evidence, the most important factor for widespread centralisation is official regulation.

## Materials and methods

### Population

Data on all patients that were diagnosed with oesophagus, pancreas and bladder cancer in The Netherlands between January 1^st^ 2000 and December 31^st^ 2013 were retrieved from the Netherlands Cancer Registry (NCR). The NCR contains patient, tumour and (hospital of) treatment data of every newly diagnosed cancer patient. Topography and morphology is coded according to the International Classification of Diseases for Oncology (ICD-O) and staging according to the TNM-classification. Quality of the data is high and completeness is estimated to be at least 95%.[19, 20] The total number of inhabitants of The Netherlands was 15.9 million in 2000 and 16.8 million in 2013.[21]

We included patients with oesophagus tumours including cardia (C15.0–15.9, C16.0), pancreas and peri-ampullary tumours (C25.0–25.9, C24.1, C17.0) and bladder tumours (C67.0–67.9). Exclusion criteria were: unknown hospital of surgery or diagnosis at autopsy. Per tumour the total annual surgical volume was calculated per hospital. In the NCR the type of surgery was not completely specified before 2005. Different types of surgery could have been coded under a non-specified surgical code; patients with the same treatment code could have had a pancreaticoduodenectomy or only local treatment. We accepted this for oesophagus and pancreas cancer because local surgical treatment was not common practice then. From 2005 we were able to differentiate oesophagus(cardia) resections and pancreatectomies. We included all types of surgery for pancreas malignancies, not only (pylorus preserving) pancreaticoduodenectomies, but also pancreas tail resections. Local surgical treatment is more common in bladder cancer, therefore the centralisation of cystectomies is studied from 2005 onwards. Only the initial treatment (within six months after diagnosis) for every new bladder tumour was registered, thereby disregarding cystectomy for an initial non muscle-invasive tumour that progressed to muscle-invasive disease more than six months after the first diagnosis and a salvage cystectomy after radiotherapy. When the initial treatment took more than six months to complete, e.g. in case neo-adjuvant chemotherapy, the cystectomy was registered.

### Analyses

Hospitals were categorised based on the surgery volume per tumour per year: <10, 10–19 and ≥20. These categories were chosen based on the first minimum annual thresholds of 10 which later changed to 20. If the year of surgery was unknown the year of incidence was used. Timelines with the proportion of patients per hospital category were plotted from 2000–2013 (cystectomies from 2005–2013) with descriptions of important influencing factors including landmark studies, regulation, and guidelines by specialists societies. STATA version 12.0 was used for the main analyses. Trendbreak was analysed using Joinpoint Software. Because the minimum surgical volume for pancreas and oesophagus cancer was 10 until 2011 and still is 10 for cystectomies we analysed trendbreak for minimum annual volumes of 10 (including the ≥10 and ≥20 category).

## Results

**Fig 1:**
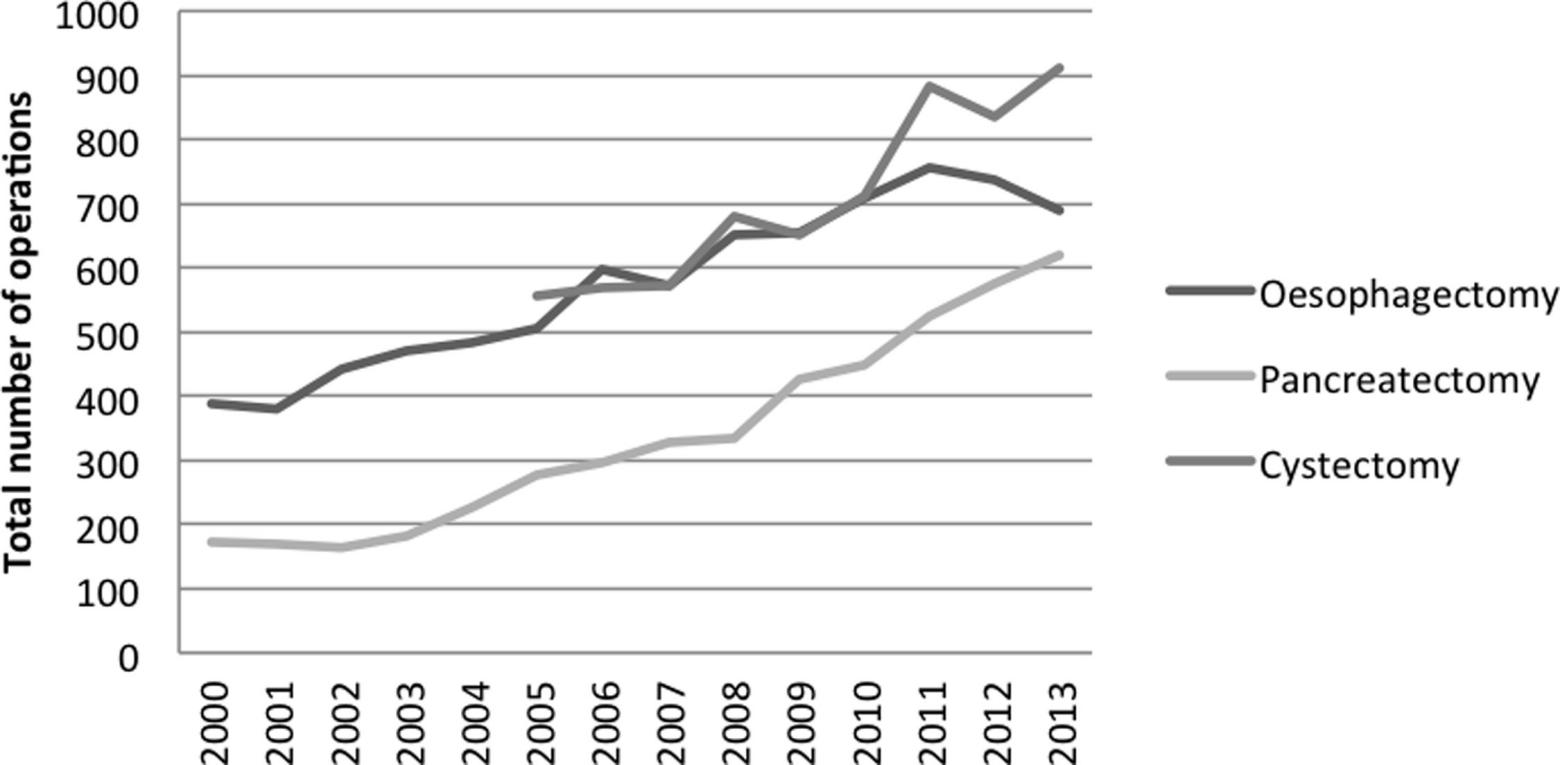
Total number of oesophagectomies and pancreatectomies from 2000–2013 and cystectomies from 2005–2013 in the Netherlands.

The study population is presented in Table 1. The high number of patients with bladder cancer can be explained by the high numbers of carcinoma in situ. Fig 1 shows an increasing number of surgical procedures for oesophagus, pancreas and bladder cancer.

**Table 1:** Characteristics of the study population of oesophagus and pancreas cancer (2000–2013) and bladder cancer (2005–2013).

	Oesophagus N (%)	Pancreas N (%)	Bladder N (%)
**Total number of patients**	29,399	19,630	52,763
**Sex**			
Male	21,557 (73.3)	10,474 (53.36)	40,820 (77.36)
Female	7,842 26.7)	9,156 (46.64)	11,943 (22.64)
**Stage**			
0	296 (1.01)	175 (0.89)	27,539 (52.20)
1	2,683 (9.13)	1,726 (8.79)	10,776 (20.42)
2	3,950 (13.44)	3,950 (20.12)	5,759 (10.91)
3	6,405 (21.79)	2,334 (11.89)	3,125 (5.92)
4	10,899 (37.07)	9,876 (50.31)	4,892 (9.27)
unknown	5,166 (17.58)	1,569 (8.00)	672 (1.27)
**Receiving surgery**			
Yes	8,037 (27.3)	4,747 (24.18)	6,362 (12.06)
No	21,362 (72.7)	14,883 (75.82)	4,6401 (87.94)

### Oesophagus cancer

**Fig 2:**
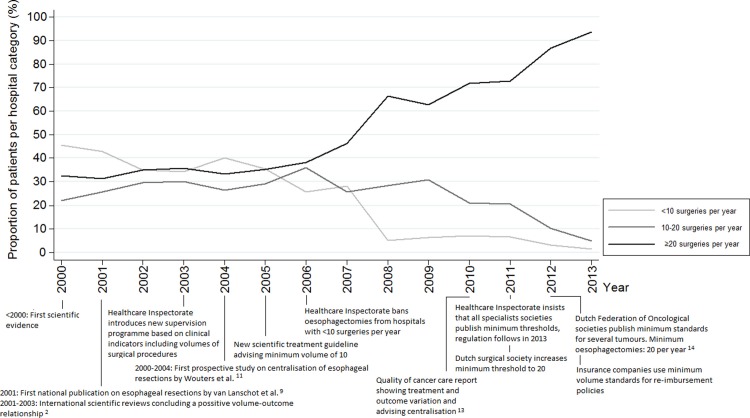
Changes in surgical volumes from 2000–2013: Oesophageal resections and relevant external stimuli. Significant trendbreak in 2005 (for cumulative categories ≥10 and ≥ 20 procedures).

The total number of operations per year gradually increased from 387 in 2000 to 690 in 2013 (Fig 1). Initially, the rise in absolute volume was not represented in the volume categories (Fig 2). Trendbreak analysis was significant in 2005 and a strong rise can be seen in the ≥20 category from 2006 onwards. In the period before 2006, the rising number of patients was distributed among all three volume categories while the rise in incidence after 2006 contributed foremost to the >20 surgeries per year category. This is represented by the total number of hospitals that performed oesophagus surgery. This varied from 55 in 2000 to 64 in 2004 and 55 in 2006. A sharp decrease was then seen in the period from 2011–2013; in 2011 there were still 43 hospitals performing oesophagus surgery, which dropped to 27 in 2013. The trendbreak in 2006 coincides with the execution of a Dutch prospective study from 2000–2004 which was published in 2009 but reported upon in national fora earlier and the new minimum threshold for oesophageal resections that was set on 10 per year by the Healthcare Inspectorate in 2006.[11]. In 2013 93% of the patients were operated in hospitals that perform 20 or more surgeries per year.

### Pancreas cancer

**Fig 3:**
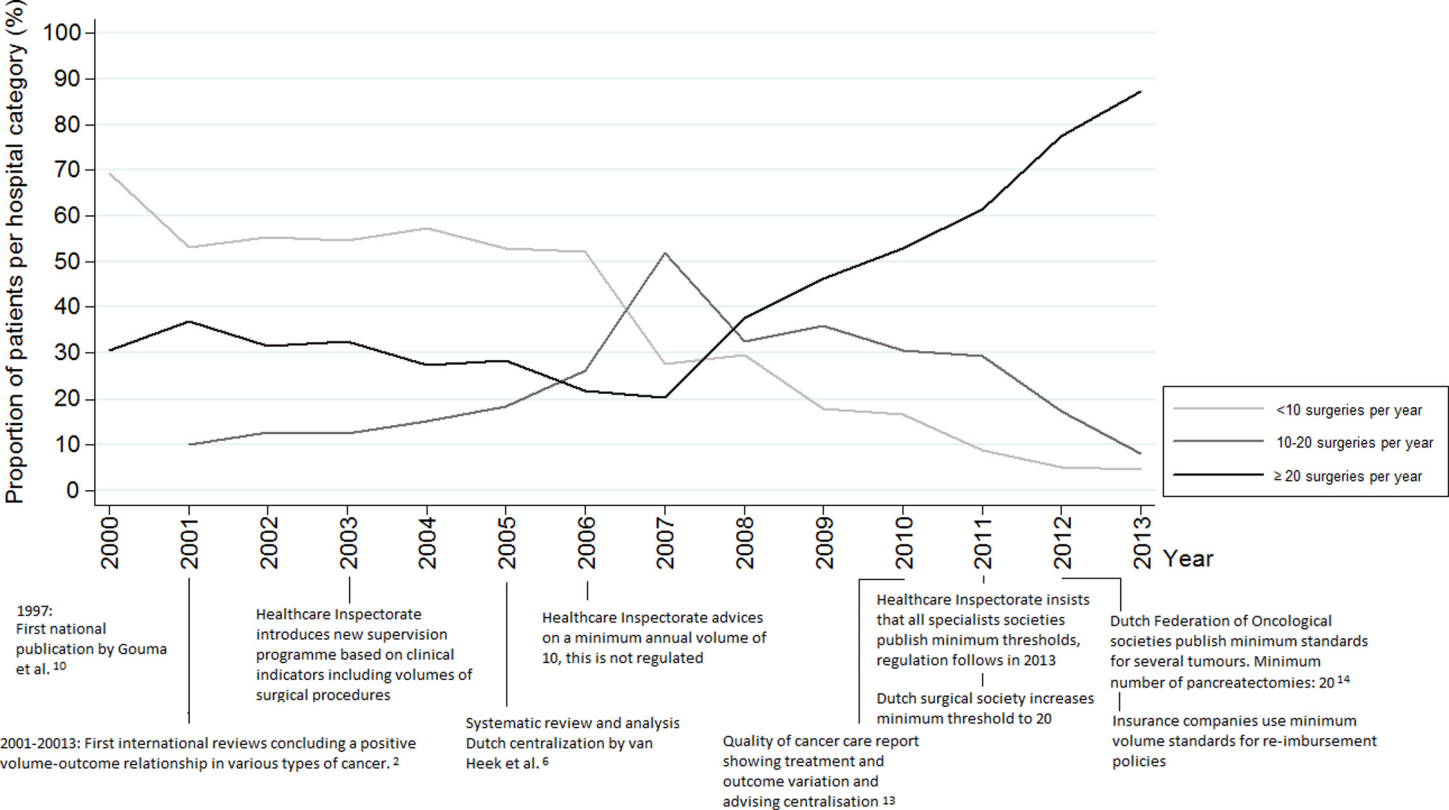
Changes in surgical volumes from 2000–2013: Pancreas resections and relevant external stimuli. Significant trendbreak in 2006 (for cumulative categories ≥10 and ≥ 20 procedures).

Fig 1 shows a sharp increase in the total volume of pancreas surgery, from 174 in 2000 to 621 in 2013. Like in oesophagus surgery, this rise in absolute volume was initially distributed among all three volume categories, with a steep rise in the 10–20 category between 2004 and 2007. Until 2011 the 0–10 and 10–20 categories remain relatively stable, implying that the extra influx of patients foremost contributed to the >20 category. The number of hospitals reflects this observation: in 2000 there were 41 hospitals that performed pancreas surgery, rising to 47 in 2007 and gradually decreasing to 38 in 2013. This is still a relatively high number of hospitals which is partially caused by our inclusion of all types of pancreas surgery. When disregarding partial resections (e.g. pancreas tail resections) and only including classical Whipple’s or PPPD’s the number of hospitals dropped from 39 in 2007 to 25 in 2013. Centralisation occurred rapidly after an initial trendbreak in 2006 and further intensified from 2011 onwards (Fig 3). In 2013 almost 90% of the patients were operated in a hospital with a yearly volume of 20 or higher.

### Bladder cancer

**Fig 4:**
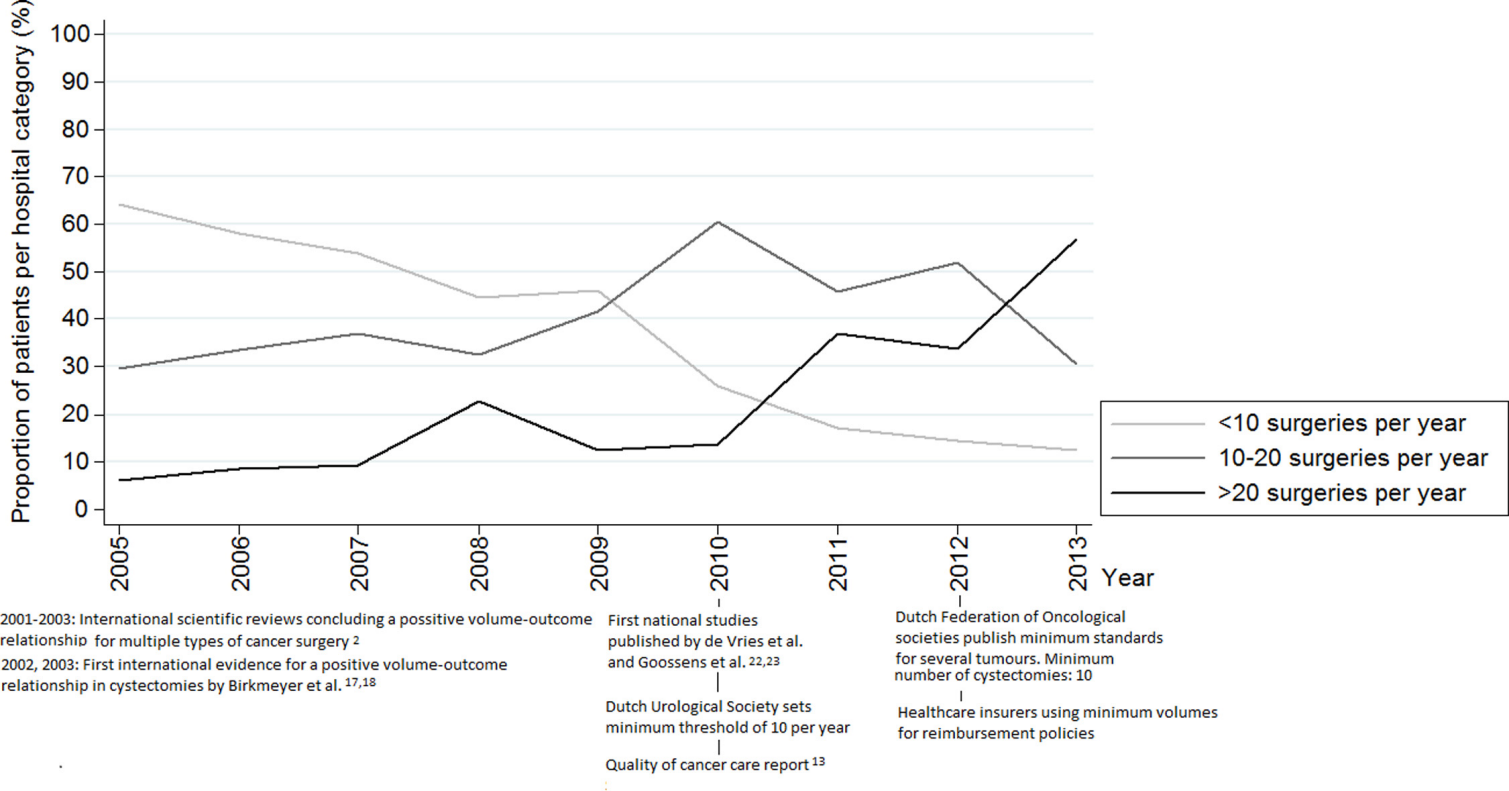
Changes in surgical volumes from 2005–2013: Cystectomies and relevant external stimuli.

Fig 4 shows a late onset of centralisation compared to oesophagus and pancreas surgery. No significant trendbreak was evident. A gradual decrease in the <10 category can be seen from 2009 onwards. A strong increase in centralisation to 20 or more surgeries per year can be seen in 2013. The absolute volume of surgery increased from 554 in 2005 to 912 in 2013 (Fig 1). In 2005 cystectomies were performed in 85 hospitals, decreasing from 80 in 2009 to 60 in 2013.

## Discussion

Our results show that centralisation started in the years following the publication of scientific evidence from Dutch studies and international reviews. Scientific evidence obviously preludes centralisation but does not seem sufficient to initiate a widespread effect. Official publication of minimum standards by the medical specialists societies intensified centralisation, especially in the years before and after publication. This can be seen in all three tumour types. Because official regulation sometimes initiated the publication of minimum standards by the specialist societies and intensified after that, there seemed to be interaction between the two phenomenon’s though regulation seems to have more impact.

Centralisation of oesophageal resections started in 2006 and from 2008 onwards more than 90% of the patients were treated in hospitals with a surgical volume ≥10 per year. A regional prospective study in the Netherlands investigated the effects of centralisation of oesophageal resections from 2000–2004. Along with a reduction in postoperative morbidity and length of stay, mortality was shown to fall from 12% to 4% and survival improved significantly. The hospitals with the highest procedural volume showed the biggest improvements in outcome.[11] The results were shared in national conferences and combined with the growing international evidence strongly enforced the centralisation of surgery for patients with oesophagus cancer. Consequently, in 2006 the Dutch Health Inspectorate set the minimum threshold on ten per year and centralisation followed rapidly. The second rise in concentration can be seen from 2011 onwards when hospitals were required to perform at least 20 resections per year which resulted in a decreasing proportion of patients treated in a hospital with an average annual volume between 10–20 (Fig 2). The total number of hospitals performing these surgeries supports the findings that a ‘true’ centralisation effect was happening, instead of an effect occurring because of the rising incidence. It seems that a combination of scientific evidence and regulation was needed to implement widespread centralisation. Whether scientific evidence alone has the same effect is questionable when looking at the centralisation pattern of the other two tumours. While trendbreak analyses for the centralisation of pancreatic cancer surgery show a significant increase from 2006 onwards, it took until 2009 for more than 80% of the patients to be operated in a hospital with an annual volume ≥10. Unlike for oesophagectomies, between 2004 and 2011 no officially regulated minimum threshold for pancreas surgery existed. The decrease that can be seen in pancreas surgery in hospitals with an annual volume <10 co-occurs with the threat of regulation. It might also have been triggered by the centralisation of oesophagus surgery. It is likely that professionals regarded pancreas surgery as a logical next step in centralisation. In bladder cancer, the process of centralisation started late compared to oesophagus and pancreas surgery. A sharp increase in centralisation can be seen in 2009, a year before the Dutch Society for Urology decided on a minimal annual cystectomy threshold of 10. This coincided with the quality of cancer care report from the Dutch Cancer Society and two Dutch studies on the effects of volume on outcomes after cystectomy. De Vries et al. observed lower post-operative mortality related to higher surgical volumes but this difference could not reach statistical significance.[22] Goossens et al. found that postoperative mortality after cystectomy is significantly inversely associated with high-volume providers.[23] Furthermore, a study published in 2012 comprising data from 2000–2008 confirmed the inverse relationship between hospital volume and mortality and morbidity in the Netherlands. The results of this study showed that the chance of undergoing cystectomy was significantly higher in high-volume hospitals. Long-term survival after cystectomy was also higher in high-volume hospitals.[24] This might explain the increase in centralisation in 2013 together with discussions on the appropriateness of the (low) threshold of 10. In January 2015 a minimum number of 20 cystectomies per year per hospital was decided upon by the Dutch urological society.[25]

For all three cancers, the impact exerted by healthcare insurers on centralization grew from 2011 onwards when they started to incorporate minimum volume standards in their reimbursement negotiations with hospitals. There was a threat that low volume hospitals would not be reimbursed in the future. This pressured hospitals to re-evaluate their position in the oncological surgical field. However, in our study period, a true ‘negative incentive’ by completely stopping reimbursements for low volume centres was not yet common practice.

The use of the population based Netherlands Cancer registry as our data source is a major strength of our study. This allowed us to analyse high volumes of patients over a long time period. Our study also had some limitations. The Netherlands Cancer Registry did not always specify the type of surgery or hospital of surgery in the period before 2005. Therefore, patients that received local surgical tumour treatment instead of extensive surgery can be present in our analyses for oesophagus and pancreas cancer. Because these therapies are not the primary treatment options we argue that the effect on our analyses is small. Furthermore, the question can be raised if any surgical procedure for oesophagus and pancreas cancer should take place in a high volume hospital anyway. Although the standards are based on malignancies, surgery for benign conditions is not registered in the NCR which may give an underestimation of the volume of surgeries in that organ. The impact of excluding patients that were treated in an unknown hospital of surgery is likely to be small, for oesophagus surgery this accounted for 14% between 2000–2005. Our study focusses on a national level and regional initiatives such as cooperation between groups of surgeons might also have influenced centralisation. In our analyses we use hospital volume, not the number of operations per surgeon. It can be argued that a high number of operations per surgeon is more important than hospital volume. Previous research was not conclusive on this issue. There are multiple studies reporting on a more important effect of hospital volume arguing that improving quality depends on multidisciplinary aspects of hospital care rather than solely on intraoperative technique. [26, 27, 28]

Our results show that international scientific evidence was not strong enough to convince large numbers of physicians to change their daily practice and centralise surgical procedures. Arguments against the generalisability to the Dutch healthcare situation were weakened by a growing body of evidence and more importantly, national studies with convincing data. Regulation did not start centralisation, but followed scientific evidence and subsequent voluntary centralisation. Strong national scientific evidence proved to be needed for acceptance in the field and in addition, regulation seems necessary to implement widespread centralisation. In contrast to ‘regular clinical cancer research’ the results of organisational change studies are likely to be greeted with more scepticism which hinders acceptance and implementation. Studies with solid designs unravelling the mechanisms of organisational aspects and choices (such as centralisation) are needed for wider acceptance in the field. In general. it seems inevitable that once a body of evidence has been established on organisational change that influences professional practice, some form of regulation needs to be added to ensure widespread implementation.

## References

[1] Luft HS, Bunker JP, Enthoven AC. Should operations be regionalized? The empirical relation between surgical volume and mortality. N Engl J Med. 1979;301(25):1364–9. doi: 10.1056/NEJM197912203012503 503167 10.1056/NEJM197912203012503

[2] Hillner BE, Smith TJ, Desch CE. Hospital and physician volume or specialization and outcomes in cancer treatment: importance in quality of cancer care. J Clin Oncol. 2000;18(11):2327–40. doi: 10.1200/JCO.2000.18.11.2327 10829054 10.1200/JCO.2000.18.11.2327

[3] Killeen SD O 'Sullivan MJ, Coffey JC, Kirwan WO, Redmond HP. Provider volume and outcomes for oncological procedures. The British journal of surgery. 2005;92(4):389–402. doi: 10.1002/bjs.4954 10.1002/bjs.4954 15786424

[4] Dudley RA, Johansen KL, Brand R, Rennie DJ, Milstein A. Selective referral to high-volume hospitals: estimating potentially avoidable deaths. Jama. 2000;283(9):1159–66. 10703778 10.1001/jama.283.9.1159

[5] Halm EA, Lee C, Chassin MR. Is volume related to outcome in health care? A systematic review and methodologic critique of the literature. Ann Intern Med. 2002;137(6):511–20. 12230353 10.7326/0003-4819-137-6-200209170-00012

[6] van Heek NT, Kuhlmann KF, Scholten RJ, de Castro SM, Busch OR, van Gulik TM, et al Hospital volume and mortality after pancreatic resection: a systematic review and an evaluation of intervention in the Netherlands. Ann Surg. 2005;242(6):781–8, discussion 8–90. doi: 10.1097/01.sla.0000188462.00249.36 16327488 10.1097/01.sla.0000188462.00249.36 PMC1409869

[7] Ho VK, van der Heiden-van der Loo M, Rutgers EJ, van Diest PJ, Hobbelink MG, Tjan-Heijnen VC, et al Implementation of sentinel node biopsy in breast cancer patients in the Netherlands. European journal of cancer. 2008;44(5):683–91. doi: 10.1016/j.ejca.2008.01.027 18314328 10.1016/j.ejca.2008.01.027

[8] Gouma DJ, van Geenen RC, van Gulik TM, de Haan RJ, de Wit LT, Busch OR, et al Rates of complications and death after pancreaticoduodenectomy: risk factors and the impact of hospital volume. Ann Surg. 2000;232(6):786–95. 11088073 10.1097/00000658-200012000-00007 PMC1421271

[9] van Lanschot JJ, Hulscher JB, Buskens CJ, Tilanus HW, ten Kate FJ, Obertop H. Hospital volume and hospital mortality for esophagectomy. Cancer. 2001;91(8):1574–8. 11301408 10.1002/1097-0142(20010415)91:8<1574::aid-cncr1168>3.0.co;2-2

[10] Gouma DJ, De Wit LT, Van Berge Henegouwen MI, Van Gulik TH, Obertop H. [Hospital experience and hospital mortality following partial pancreaticoduodenectomy in The Netherlands]. Nederlands tijdschrift voor geneeskunde. 1997;141(36):1738–41. 9545716

[11] Wouters MW, Karim-Kos HE, le Cessie S, Wijnhoven BP, Stassen LP, Steup WH, et al Centralization of esophageal cancer surgery: does it improve clinical outcome? Annals of surgical oncology. 2009;16(7):1789–98. doi: 10.1245/s10434-009-0458-9 19370377 10.1245/s10434-009-0458-9 PMC2695873

[12] Smolders KH, Den Ouden AL, Nugteren WA, Van Der Wal G. Does public disclosure of quality indicators influence hospitals' inclination to enhance results? Int J Qual Health Care. 2012;24(2):129–34. doi: 10.1093/intqhc/mzs003 22315017 10.1093/intqhc/mzs003

[13] Wouters MW, Jansen-Landheer ML, van de Velde CJ. The Quality of Cancer Care initiative in the Netherlands. Eur J Surg Oncol. 2010;36 Suppl 1:S3–S13. 20576399 10.1016/j.ejso.2010.06.004

[14] Stichting Oncologische Samenwerking. Multidisciplinaire normering oncologische zorg Nederland. 2015. Available from: www.soncos.org

[15] Elting LS, Pettaway C, Bekele BN, Grossman HB, Cooksley C, Avritscher EB, et al Correlation between annual volume of cystectomy, professional staffing, and outcomes: a statewide, population-based study. Cancer. 2005;104(5):975–84. doi: 10.1002/cncr.21273 16044400 10.1002/cncr.21273

[16] Hollenbeck BK, Daignault S, Dunn RL, Gilbert S, Weizer AZ, Miller DC. Getting under the hood of the volume-outcome relationship for radical cystectomy. The Journal of urology. 2007;177(6):2095–9; discussion 9. doi: 10.1016/j.juro.2007.01.153 17509295 10.1016/j.juro.2007.01.153

[17] Birkmeyer JD, Siewers AE, Finlayson EV, Stukel TA, Lucas FL, Batista I, et al Hospital volume and surgical mortality in the United States. N Engl J Med. 2002;346(15):1128–37. doi: 10.1056/NEJMsa012337 11948273 10.1056/NEJMsa012337

[18] Birkmeyer JD, Stukel TA, Siewers AE, Goodney PP, Wennberg DE, Lucas FL. Surgeon volume and operative mortality in the United States. N Engl J Med. 2003;349(22):2117–27. doi: 10.1056/NEJMsa035205 14645640 10.1056/NEJMsa035205

[19] Schouten LJ, Hoppener P, van den Brandt PA, Knottnerus JA, Jager JJ. Completeness of cancer registration in Limburg, The Netherlands. International journal of epidemiology. 1993;22(3):369–76. 8359950 10.1093/ije/22.3.369

[20] Schouten LJ, Jager JJ, van den Brandt PA. Quality of cancer registry data: a comparison of data provided by clinicians with those of registration personnel. British journal of cancer. 1993;68(5):974–7. 8217612 10.1038/bjc.1993.464 PMC1968711

[21] CBS Statistics Netherlands. 2015 [cited 2015 September 20th]. Available from: http://statline.cbs.nl/StatWeb/publication/?VW=T&DM=SLNL&PA=37296ned&D1=a&D2=0,10,20,30,40,50,60,(l-1),l&HD=130605-0924&HDR=G1&STB=T

[22] de Vries RR, Visser O, Nieuwenhuijzen JA, Horenblas S, Members of the Urological Oncology Working Group of the Comprehensive Cancer Centre A. Outcome of treatment of bladder cancer: a comparison between low-volume hospitals and an oncology centre. World journal of urology. 2010;28(4):431–7. doi: 10.1007/s00345-010-0512-z 20130885 10.1007/s00345-010-0512-z

[23] Goossens-Laan CA, Visser O, Wouters MW, Jansen-Landheer ML, Coebergh JW, van de Velde CJ, et al Variations in treatment policies and outcome for bladder cancer in the Netherlands. Eur J Surg Oncol. 2010;36 Suppl 1:S100–7. 20598491 10.1016/j.ejso.2010.06.003

[24] Goossens-Laan CA, Visser O, Hulshof MC, Wouters MW, Bosch JL, Coebergh JW, et al Survival after treatment for carcinoma invading bladder muscle: a Dutch population-based study on the impact of hospital volume. BJU international. 2012;110(2):226–32. doi: 10.1111/j.1464-410X.2011.10694.x 22044615 10.1111/j.1464-410X.2011.10694.x

[25] Dutch Society for Urology. Kwaliteitsnormen Blaascarcinoom. 2015 Januari 2015.

[26] Billingsley KG, Morris AM, Dominitz JA, Matthews B, Dobie S, Barlow W, Wright GE, Baldwin L. Surgeon and Hospital Characteristics as Predictors of Major Adverse Outcomes Following Colon Cancer SurgeryUnderstanding the Volume-Outcome Relationship. Arch Surg. 2007;142(1):23–31. doi: 10.1001/archsurg.142.1.23 17224497 10.1001/archsurg.142.1.23

[27] Liu C.-J., Chou Y.-J., Teng C.-J., Lin C.-C., Lee Y.-T., Hu Y.-W., Yeh C.-M., Chen T.-J. and Huang N. (2015), Association of surgeon volume and hospital volume with the outcome of patients receiving definitive surgery for colorectal cancer: A nationwide population-based study. Cancer, 121: 2782–2790. doi: 10.1002/cncr.29356 25892632 10.1002/cncr.29356

[28] Schrag D., Panageas K. S., Riedel E., Hsieh L., Bach P. B., Guillem J. G. and Begg C. B. (2003), Surgeon volume compared to hospital volume as a predictor of outcome following primary colon cancer resection. J. Surg. Oncol., 83: 68–78. doi: 10.1002/jso.10244 12772198 10.1002/jso.10244

